# Clinical implications of recurrent gene mutations in acute myeloid leukemia

**DOI:** 10.1186/s40164-020-00161-7

**Published:** 2020-03-27

**Authors:** Jifeng Yu, Yingmei Li, Danfeng Zhang, Dingming Wan, Zhongxing Jiang

**Affiliations:** 1 Department of Hematology, The First Affiliated Hospital of Zhengzhou University, Zhengzhou, 450052 Henan China; 2 Academy of Medical and Pharmaceutical Sciences, Zhengzhou University, Zhengzhou, 450052 Henan China

**Keywords:** Recurrent gene mutation, Acute myeloid leukemia (AML), FLT3, CEBPA, NPM1, RUNX1, ASXL1, TP53, IDH1/2

## Abstract

Acute myeloid leukemia (AML) is a genetically heterogeneous clonal malignancy characterized by recurrent gene mutations. Genomic heterogeneity, patients’ individual variability, and recurrent gene mutations are the major obstacles among many factors that impact treatment efficacy of the AML patients. With the application of cost- and time-effective next-generation sequencing (NGS) technologies, an enormous diversity of genetic mutations has been identified. The recurrent gene mutations and their important roles in acute myeloid leukemia (AML) pathogenesis have been studied extensively. In this review, we summarize the recent development on the gene mutation in patients with AML.

## Introduction

Acute myeloid leukemia (AML) is a genetically heterogeneous clonal malignancy originating from clonal hematopoietic stem-cells, characterized by chromosomal abnormalities, recurrently gene mutations, epigenetic modifications affecting chromatin structure, and microRNAs deregulations. Genomic heterogeneity, patients’ individual variability, and recurrent gene mutations are the few major obstacles among many factors that impact treatment efficacy of the AML patients [1, 2].

With the application of new molecular techniques, such as cost- and time-effective next-generation sequencing (NGS) technologies, an enormous diversity of genetic mutations has been identified. Six genes, including FMS-like tyrosine kinase 3 (FLT3), nucleophosmin 1 (NPM1), CCAAT/enhancer binding protein alpha (CEBPA), Runt-related transcription factor 1 (RUNX1), additional sex combs-like 1 (ASXL1), and tumor protein p53 (TP53), have already been incorporated into the risk categories proposed by the European Leukemia Net (ELN) [2]. Other recurrent gene mutations have been reported in AML patients [3–8]. Furthermore, the important roles of recurrent gene mutation in AML pathogenesis had been explored and gene mutation-targeted therapies had been developed [8–15]. Certain genes have been proved to be specifically related to the leukemia pathogenesis, such as pre-leukemic cell identification, particularly in AML patients with mutated DNMT3A and TET2 [16, 17]. DNMT3A and TET2 are common mutated genes in patients with clonal hematopoiesis of indeterminate potential (CHIP) [18–21] and it might be considered as preleukemia cells identification markers [16, 17].

In this review, we summarize the recent development on the clinical implications of recurrent gene mutations in patients with AML.

### FLT3

FLT3 is a type III receptor tyrosine kinase that plays an important role in hematopoietic cell survival, proliferation and differentiation. The important clinical point is that mutation of the FLT3 gene is the most frequent genetic alteration and a poor prognostic factor in AML patients. Mutations of the FLT3 gene occur in approximately 30% of all AML cases. There are two major types of FLT3 mutations: internal tandem duplication (FLT3-ITD) mutations in the juxtamembrane domain, which represents the most common type of FLT3 mutation occurring in approximately 25% of all AML cases, and point mutations or deletion in the tyrosine kinase domain (FLT3-TKD) occurring in approximately 7–10% of all cases with prognostic value uncertain [22–26]. Both mutant FLT3 molecules are activated through ligand-independent dimerization and trans-phosphorylation. Patients with FLT3-ITD have a high risk of relapse and low cure rates [22–25]. Risk associated to FLT3-ITD in patients with AML may depend on mutational burden and its interaction with other mutations. Allogeneic stem cell transplantation in first complete remission (CR1) was associated with a reduced relapse risk in all molecular subgroups with the exception of NPM1mut AML with absent or low ratio FLT3-ITD [27]. Another study showed patients with co-mutated NPM1 and FLT3-TKD may have an exceptionally favorable prognosis [28]. ELN-2017 guidelines recommend upfront testing for FLT3 and measurement of allele ratio (AR) for the prognosis risk stratification. For those patients with FLT+ testing, it’s important to incorporate targeted FLT3 inhibitors into the therapy regimen to improve the patients’ outcome [2]. Although FLT3–ITD–AR can be used for selecting patients for treatment with TKIs, but a relevance of ITD length may be the indicator for both outcome and response to FLT3-inhibitors. FLT3-ITD-AR on RNA measurement is recommended because superior prognostic value and accurate mRNA length measurement [2, 12]. Increasing FLT3-ITD AR and insertion site in the TKD1 were associated with low CR rates [23]. The insertion site was strongly correlated with ITD size: more C-terminal located inserted fragments were significantly bigger. A high mutant/wild-type ratio appears to have a major impact on the prognostic relevance [29]. Patients with more than one ITD had a significantly shorter OS and RFS [30].

Retrospective validation study of the ELN-2017 guidelines on the classification for AML with NPM1 and FLT3-ITD genotypes demonstrated that the ELN-2017 was more accurate to distinguish prognosis in patients with newly diagnosed AML [25]. However, when comparing patients with a low FLT3-ITD AR with those with a high FLT3-ITD AR, no significant differences in survival were noted in FLT3+ patients irrespective of NPM1 mutational status regardless of whether they were treated with intensive chemotherapy with or without a FLT3 inhibitor—a result that is discordant from the current ELN guidelines [25].

Other studies have demonstrated patients with mutations of FLT3-ITD, DNA methyltransferase 3A (DNMT3A), isocitrate dehydrogenases (IDH) 1, and ten-eleven translocation 2 (TET2) were risk factors for overall survival (OS) [4, 26]. The mutation patterns of Chinese AML patients by NGS revealed correlations between gene mutations and clinical features. Routine testing of suspected genes by NGS are recommended for better prognostic prediction and individualized treatment [4]. FLT3-ITD mutational status and KMT2E gene expression levels can be used to identify those AML patients who need to be treated differently to maximize their chances of cure [31]. RNA-based FLT3-ITD measurements are recommended for risk stratification, and the relevance of AR regarding eligibility for FLT3-targeted therapy remains uncertain [12].

Recent study demonstrates that PRMT1-mediated FLT3 methylation promotes AML maintenance and suggests that combining PRMT1 inhibition with FLT3 tyrosine kinase inhibitors treatment could be a promising approach to eliminate FLT3-ITD+ AML cells [32]. FLT3-TKD can activate the downstream effector molecule signal transducer and activator of transcription 5 (STAT5) exclusively in the presence of mutated NPM1c. NPM1c mislocalizes FLT3-TKD and changes its signal transduction ability [33].

Among those recurrent gene mutations, the most adverse prognosis was observed in patients with DNMT3A and FLT3-ITD co-mutation, whose survival could be significantly improved with allo-HSCT [7, 34]. Recurrent gene mutations analysis showed JAK2, FLT3-ITD^high^, and KIT^high^ mutations were identified as significant prognostic factors for OS in multivariate analysis [35]. FLT3-ITD mutant AR is closely related to CR and OS in AML patients. Classifying risk grades based on FLT3-ITD mutant AR is crucial for individualized treatment and prognostic evaluation [36]. Targeting mutant FLT3 by small molecule inhibitors has rapidly emerged as a new therapeutic approach in patients with AML. Different FLT3 inhibitor therapeutic agents have been developed and are summarized in Tables 1, 2 [9, 37–53].

**Table 1:** Gene mutation frequency and clinical implications

Gene name	Function of native genes/proteins	Mutant frequency in AML	Mutant gene clinical significance
FLT3	FLT3 gene located on chromosome 13 A receptor tyrosine kinase Exclusively in hematopoietic compartment Mediates HSC survival, proliferation and differentiation	FLT3 mutation ~ 30% of all AML FLT3-ITD ~ 25% of AML FLT3-TKD ~ 7–10% of AML	Associated with R/R AML and poor OS Favorable prognosis co-mutation NPM1 with FLT3-TKD ELN guidelines recommend upfront testing for FLT3 and AR Recommend to use targeted FLT3 inhibitors to improve outcomes
NPM1	NPM1 gene located on chromosome 5 Multifunctional phosphoprotein in the granular portion of nucleolus Regulates multiple cellular events Regulates key tumor suppressor proteins ARF, p53 and MDM2	~ 30% of all AML 40–60% with normal karyotypes	Favorable mutant Higher CR rate Improved OS Lower cumulative incidence of relapse A stable marker for assessment of MRD
CEBPA	CEBPA gene located on chromosome 19 A transcription factor Strongly implicated in myelopoiesis Control proliferation and differentiation of myeloid progenitors	10–20% of AML with normal karyotypes ~ 50% with biCEBPA	WHO recognizes biCEBPA as a unique entity Favorable prognosis of biCEBPA mutations with standard therapy biCEBPA more likely co-mutate with TET2 and GATA2 moCEBPA more likely co-mutate with NPM1, FLT-3 ITD/TKD, and IDH2
RUNX1	RUNX1 gene located on chromosome 21 Regulate critical processes in hematopoiesis Define definitive hematopoietic stem cell	~ 10–15% of all AML	Involved in t(8;21) in AML Fusion protein between RUNX1 and ETO A new entity “AML with mutated RUNX1” Associated with inferior outcome Co-mutation DNMT3A and RUNX1 with inferior OS in age < 60 AML
ASXL1	ASXL1 gene located on chromosome 20q11 An epigenetic modulator Mutant ASXL1 protein distracts hematopoiesis and promotes myeloid transformation by altering histone modifications	~ 15–20% of all AML 5 times more common in older patients (≥ 60 years) than younger patients (< 60 years)	Associated with inferior OS More coexist with RUNX1, IDH2 Clonal marker for R/R AML
TP53	TP53 genes located on chromosome 17p Critical role in tumor suppression Mutation/deletion causes different tumors	< 10% of de novo AML 20–37% of patients with sAML/tAML ~ 70% of patients with a complex karyotype Prevalent with R/R AML	Poor OS with standard therapy More frequent in older patients More frequent with 17p mutations More frequent with aberrations in chromosomes 5 and 7 More likely in R/R AML

**Table 2:** Molecular target therapeutic agents

Target	Types	Agents	Clinical efficacy
FLT3 inhibitors	Type 1	Midostaurin	4-year OS 51.4% on midostaurin versus 44.2% on placebo on FLT3+ AML [37] EFS and OS at 2 years were 39% and 34% in younger and 53% and 46% in older patients, respectively [38] Recommended as front line therapy for AML with FLT3-ITD and FLT3-TKD [9, 39]
		Sunitinib	Some promising results in phase I/II clinical trials, but with high incidence of adverse effects [40, 41]
		Gilteritinib^a^	R/R FLT3-mutated AML, median OS for single agent gilteritinib was significantly longer than chemotherapy (9.3 months vs. 5.6 months). Median EFS was 2.8 months in the gilteritinib group and 0.7 months in chemotherapy group [42] Recommended as new standard therapy for R/R FLT3-mutated AML [43]
		Lestaurtinib	Failed to demonstrate any overall clinical benefit in a phase III trial when combined with intensive chemotherapy in patients with newly diagnosed FLT3-ITD-mutated AML [9, 44, 45]
		Crenolanib^a^	Combine with 7 + 3 regimen can overcome the poor prognostic implication of adverse mutations co-occurring with mutated FLT3 [46] Incorporation of crenolanib into frontline intensive chemotherapy resulted high ORR and may replace Midostaurin in the upfront setting [47]
	Type 2	Quizartinib^a^	Showed efficacy in multiple clinical trials in R/R AML with FLT3-ITD mutation [48–50] Recommended for patients with rapidly proliferative disease and very poor prognosis [51]
		Sorafenib	Combine with intensive chemotherapy improves OS in newly diagnosed, FLT3-ITD mutated AML regardless allogeneic HSCT [52] Improved OS for FLT3-ITD AML relapsing after allo-HSCT [53]
IDH inhibitors	IDH1	Ivosidenib	9/14 IDH1 mutation AML achieved CR + CRh (5/10 CR, 4/4 CRh) [85] Substantial efficacy with a small group study (n = 12) [86]
	IDH2	Enasidenib	ORR 38% in R/R AML [87] ORR 30.8% in older adults AML with IDH mutation. Median OS was 11.3 months [87] Benefit older adults with newly diagnosed IDH2-mutant AML who are not candidates for cytotoxic regimens [88] Fatal adverse effects including indirect hyperbilirubinemia and cytokine storm [89, 90]
	IDH1	Olutasidenib	Single agent ORR of 41% and combine with azacitidine ORR of 46% Induced deep responses with IDH1 mutation clearance [91]

^a^Second generation of FLT3 inhibitors

### TP53

Mutations in transcription factor TP53 is important in cell cycle arrest for DNA mismatch repair, base excision repair, and nucleotide excision repair. TP53 gene mutation are present in < 10% of patients with de novo AML, 20–37% of patients with sAML/tAML, and up to 70% of patients with a complex karyotype. They are increasingly prevalent with relapsed/refractory (R/R) AML patients [54–56]. TP53 mutations are more frequent in older patients with a much lower complete remission rate comparing with patients without TP53 mutations. However, the dismal response to therapy appears irrespective of age, and is associated with decreased OS regardless of therapy [56]. Part of the dismal prognosis with TP53 mutations is in part due to the resistance to chemotherapy amongst this population [56]. Recent study showed that TP53 clearance at the time of allo-SCT was predictive of better outcomes in patients who had frontline hypomethylating agent therapy. For patients with persistent TP53 at the time of allo-SCT, those received myeloablative conditioning regimen experienced worse outcomes compared to reduced intensity conditioning regimen [57]. Residual TP53 mutation contributes to chemoresistance through clonal expansion in AML. Romidepsin itself or combined with others chemotherapy drugs can potentially cure or prevent residual p53 mutation caused chemoresistance and relapse in patient [58]. Targeting the TP53 pathway in addition to novel emerging therapeutics and immunotherapy-based approaches hold promise for treatment of TP53 mutant AML [59].

### CEBPA

Transcription factor CCAAT/enhancer binding protein alpha (CEBPA) gene mutations have been found in approximately 10–20% of patients with cytogenetically normal AML, of which 50% with biallelic mutations (biCEBPA—in both N-terminal and C-terminal domains on separate alleles) [35, 60–62]. BiCEBPA has been recognized as a unique entity and recommended by ELN-2017 guidelines for upfront screening as favorable prognosis indicator with standard therapy [2, 35, 61, 62], and associated with improved event-free survival (EFS) and OS compared with all AML patients [40]. This benefit is notably exclusive to biCEBPA mutations patients, not to patients with single or monoallelic CEBPA (moCEBPA) genotypes [35, 61, 63]. CEBPA mutants have clonal heterogeneity as patients with biCEBPA are more likely to have co-mutations in TET2 and GATA2 (seen in ~ 30% of biCEBPA patients) [60, 63]. However, moCEBPA patients have a higher frequency of co-mutations in NPM1, FLT-3 ITD/TKD. IDH2 and TET2 mutations appear to confer an inferior prognosis amongst biCEBPA patients [60]. While CEBPA is currently not recommended as a marker of minimal residue disease (MRD), a specific leukemia-associated immunophenotype associated with biCEBPA mutations may serve as a valuable tool for screening and disease monitoring [64].

A recent large study on CEBPA mutations in pediatric AML showed that patients with a bZip mutation, regardless of single mutation vs. double mutations status, have a favorable prognosis. Furthermore, the study confirmed the significant overlap of CEBPA and CSF3R mutations, and demonstrated that CEBPA+/CSF3R+ patients were at high risk for relapse and thus should not be considered a low risk cohort. Given the poor outcomes with standard chemotherapy regimens, patients with CEBPA+/CSF3R+ mutations could be considered for addition of tyrosine kinase inhibitors with upfront therapy [65]. Flow cytometry detection of MRD may better predict the outcome rather than mutations based on NGS in AML with biCEBPA [63]. The WHO classification of AML with biCEBPA has been modified to require biallelic mutation of this gene, because only the biallelic mutation group is associated with an improved prognosis when compared to other AML types [66, 67]. This revised category is now considered a full entity, and supersedes AML with myelodysplasia-related changes in de novo cases [68]. Based on lineage restriction analysis, TET2 mutations could be detected in myeloid and B cells, but not T cells. DNMT3A mutations can be detected in myeloid, B and T cells in individuals with CHIP [21]. TET2 mutations have a bias towards myeloid proliferation, while DNMT3A mutations occur in multipotent stem cells. Both TET2 and CEBPA gene mutations had been used for the preleukemic cells identification [16, 17, 20, 21]. Additionally, germline CEBPA mutation has been identified in 4–15% of CEBPA double mutation AML and associated with the development of familial AML. A new category of “myeloid neoplasms with germline predisposition” has been provided by the new WHO classification and adopted by 2017 ELN Recommendations [2].

### NPM1

NPM1 is frequently mutated and the incidence occur in up to 30% of AML patients overall and 40–60% of patients with normal karyotypes [3, 4, 60, 69]. Mutated NPM1 (mNPM1) is associated with a higher complete remission, improved OS, and a lower cumulative incidence of relapse [60]. ELN-2017 guidelines incorporate NPM1 screening at diagnosis given its favorable prognostication, particularly in the absence of co-occurring transmembrane FLT3-ITD mutations with a high AR [2]. Co-occurring mutations in FLT3-ITD occur approximately twice as often in patients with mNPM1 compared with wild type (wt) NPM1 (40.2% vs. 13.7%) [2, 70]. mNPM1 is also observed in approximately 60–70% of cases with DNTM3A mutations [70, 71]. NPM1 appears to be a stable marker for assessment of MRD [72]. NPM can also be involved in leukemogenic translocation including the t(3;5)(q25;q34) NPM–MLF1 translocation, which is associated to poor clinical course but remains undefined. Recent study finds NPM and the leukemogenic NPM–MLF1 play central role in chromatin organization and gene regulation in hematopoietic cells. Results suggest that the abnormal gene regulation forced by NPM–MLF1 is different than the loss of nuclear function imposed by NPMc+, and it can be characterized by the enhanced recruitment of CHD4/NuRD to genes. Thus, NPM–MLF1 is likely to promote hematopoietic malignancies by disruption of gene regulation imposed by the NuRD activity [10].

High NPM1 variant allele frequency (VAF) is correlated with shortened OS and EFS compared to the other NPM1 mutated cases. In both univariate and multivariable analyses, high NPM1 VAF had a particularly adverse prognostic effect in the subset of patients treated with stem-cell transplantation in first remission and in patients with mutated DNMT3A, indicating the prognostic effect of NPM1 mutation in de novo AML may be influenced by the relative abundance of the mutated allele [11]. Older patients with NPM1 mutated AML have distinctive genomic mutation landscape associated with enrichment in immunosuppressive gene signature [73]. NPM1/DNMT3A and NPM1/SRSF2 as additional mutation combinations might be useful for further group refinement [74].

### RUNX1

RUNX1 is a transcription factor regulating critical processes in many aspects of hematopoiesis and is integral in defining the definitive hematopoietic stem cell. Located on chromosome 21, the RUNX1 gene is involved in many forms of chromosomal translocations in leukemia. t(8;21) is one of the most common chromosomal translocations found in AML, where it results in a fusion protein between RUNX1 and ETO. The RUNX1/ETO fusion protein is found in approximately 12% of all AML patients [75]. RUNX1/ETO maintains leukemia by promoting cell cycle progression and identifies G1 CCND-CDK complexes as promising therapeutic targets for treatment of RUNX1/ETO-driven AML [76]. Behaving mostly as loss-of-function mutations, they confer relative resistance to standard chemotherapy and are associated with unfavorable prognosis in AML [77].

As a core-binding factor of leukemia, RUNX1-RUNX1T1 had been recognized in AML and named as a new provisional entity “AML with mutated RUNX1” (excluding cases with myelodysplasia-related changes). RUNX1 mutation has been associated with distinct clinico-pathologic features and inferior outcome [2]. Mutations in DNMT3A and RUNX1 were associated only with inferior survival in younger (age < 60) patients [70].

Meta-analysis results showed that evidence supports clinical implications of RUNX1 mutations in the development and progression of AML cases and points to the possibility of a distinct category within the newer WHO classification. The findings suggest that the RUNX1 status can contribute to risk-stratification and decision-making in the management of AML [78]. Chemogenomic landscape of RUNX1-mutated AML reveals importance of RUNX1 allele dosage in genetics and glucocorticoid sensitivity. Results show the profound impact of RUNX1 allele dosage on gene expression profile and glucocorticoid sensitivity in AML, which may lead to drug repurposing and improved disease characterization [79].

### IDH1/2

IDHs are enzymes involved in multiple metabolic and epigenetic cellular processes. Mutations in IDH1 or IDH2 are detected in approximately 20% of patients with AML and induce amino acid changes in conserved residues resulting in neomorphic enzymatic function and production of an oncometabolite, 2-hydroxyglutarate. This leads to DNA hypermethylation, aberrant gene expression, cell proliferation and abnormal differentiation. IDH mutations diversely affect prognosis of patients with AML based on the location of the mutation and other co-occurring genomic abnormalities. Recently, novel therapies specifically targeting mutant IDH have opened new avenues of therapy for these patients [80].

Recent work by flow cytometry and sequencing data has identified a distinct immunophenotypic subset of NPM1-mutated AML with TET2 or IDH1/2 mutations and improved outcome. This subset of NPM1-mutated AML was associated with longer relapse-free and OS, when compared with cases that were positive for CD34 and/or HLA-DR [81].

Targeting mutant IDH by small molecule inhibitors is a rapidly emerging therapeutic approach shown by the recent approval of the first selective mutant IDH2 inhibitor, enasidenib, for the treatment of IDH2-mutated AML [82]. Using mutant isocitrate dehydrogenase as a therapeutic drug target, selecting pan-mutant IDH1/2 inhibitors in clinical trials and other mutant IDH inhibitors are under development [82, 83]. In patients with advanced IDH1-mutated R/R AML, ivosidenib demonstrated durable remissions, and molecular remissions in some patients with complete remission [84]. Different IDH inhibitor therapeutic agents are summarized in Table 2 [85–90].

### DNMT3A

DNMT3A is a kind of methyltransferase that is responsible for the de novo methylation of CpG dinucleotides. DNMT3A is crucial for the establishment and maintenance of cellular methylation patterns. The majority of the variants (approximately two-thirds of the cases) are located at R882 in exon 23. Study results have demonstrated that the DNMT3A R882 mutation disrupts the normal ligation of methyltransferase protein subunits, causing a dominant negative impact on DNMT3A protein function [91, 92]. Multiple researchers have confirmed that DNMT3A is frequently mutated in AML patients (13.5–23%) [3, 93, 94]. DNMT3A plays a unique role in hematopoiesis and AML pathogenesis as the inferior prognostic markers for AML patients [6, 95]. For other non-R882 mutations, especially for DNMT3A frameshift mutations, the occurrence is much less frequent and its prognostic significance is poorly understood [6]. AML patients with DNMT3A truncating mutations have comparable prognoses to those of DNMT3A wild type patients [96]. Distinct microRNA expression pattern for DNMT3A R882 AML patients might not only act as markers to predict disease prognosis, but also could be further investigated to develop novel therapeutic targets for patients with DNMT3A mutations [6].

DNMT3A and IDH2 mutations are synergistic events in leukemogenesis. Hematopoietic stem and progenitor cells carrying both mutations are sensitive to induced differentiation by the inhibition of both prostaglandin synthesis and HDAC [97]. However, the mutations in DNMT3A, commonly found in adults, were conspicuously absent from virtually all pediatric cases. This suggested the need for age-tailored targeted therapies for the treatment of pediatric AML [98]. Furthermore, a novel DNMT inhibitor, guadecitabine demonstrated improved pharmacokinetics and clinical activity in a subset of R/R AML patients [5].

### KMT2A

AML patients with partial tandem duplications (PTDs) in the Mixed Lineage Leukemia (MLL) officially known as the Lysine (K)-specific methyltransferase 2A (KMT2A) gene, generally have adverse outcomes. KMT2A-PTDs occur in 3.2 to 11% of adult de novo AML and are more frequently present in AML with normal cytogenetics and AML with trisomy of chromosome 11 as a sole cytogenetic aberration [99, 100]. KMT2A-PTD alone appears insufficient to cause AML and additional genetic hits are required for the development of KMT2A-PTD leukemia [101, 102]. Homogeneously staining region on chromosome 11 is highly specific for KMT2A amplification in AML and MDS [103]. Molecular landscape analysis of KMT2A-PTD AML showed the specific HOX gene expression signatures. Concurrent DNMT3A mutations and NRAS mutations are associated with an adverse outcome [100]. Cytogenetic and molecular genetic characterization of KMT2A-PTD positive AML in comparison to KMT2A-rearranged AML demonstrated both KMT2A rearrangement (KMT2Ar) and KMT2A-PTD subtypes had unfavorable outcome, particularly in patients > 60 years. Patients with KMT2Ar were younger compared to patients with KMT2A-PTD and had a higher rate of additional cytogenetic abnormalities. In both groups, occurrence of additional cytogenetic abnormalities did not influence the OS [8].

### ASXL1

As an epigenetic regulator, ASXL1 is one of the most frequently mutated genes in all subtypes of myeloid malignancies. ASXL1 mutations are also frequently detected in clonal hematopoiesis, which is associated with an increased risk of mortality [2, 104, 105]. ASXL1 mutations were associated with specific clinical and cytogenetic profiles of AML patients, such as older age, s-AML and higher peripheral leukocytosis, more frequent co-occurrence of ASXL1 mutations with trisomy 8 and chromosome 11 aberrations [106–108]. The VAF of ASXL1 and other mutations is associated with worse prognosis in patients with newly diagnosed AML [109].

Studies using ASXL1-depleted human hematopoietic cells and ASXL1 knockout mice have shown that deletion of wild-type ASXL1 protein leads to impaired hematopoiesis and accelerates myeloid malignancies via loss of interaction with polycomb repressive complex 2 proteins. On the other hand, ASXL1 mutations in myeloid neoplasms typically occur near the last exon and result in the expression of C-terminally truncated mutant ASXL1 protein [110]. There is also growing evidence indicating that the physiological expression of mutant ASXL1 protein perturbs hematopoiesis and promotes myeloid transformation by altering histone modifications in both dominant-negative and gain-of-function manners [111].

ASXL1 mutations in AML are more frequently coexist with RUNX1, IDH2 and other mutations [107, 108]. Notably, RUNX1 is the most frequently mutated gene in ASXL1-mutated AML. Coexistence of ASXL1 and RUNX1 mutations is related to poor prognosis in AML patients [107]. ASXL1 somatic mutation exists in hematological neoplasms including MDS, AML, MPN and MDS/MPN, and often is associated with somatic mutations of TET2, EZH2, IDH2, RUNX1, NRAS and DNMT3A. The pattern of clonal evolution suggests that this ASXL1 mutation might be an early mutational event that occurs in the principal clonal population and can serve as a clonal marker for R/R disease [112].

Mutations in genes encoding epigenetic modifiers, such as DNMT3A, ASXL1, TET2, IDH1, and IDH2, are commonly acquired early and are present in the founding clone. By contrast, mutations involving NPM1 or signaling molecules (e.g., FLT3, RAS gene family) are typically secondary events that occur later during leukaemogenesis [113, 114]. Novel promising therapeutic strategies had been established for targeting ASXL1 mutated myeloid malignancies by blocking interactions between ASXL1 and associating epigenetic regulators [115–118]. Furthermore, recurrent somatic mutations in more than 50 genes have been identified in 80–90% of MDS. The most recurrent genetic mutations are involved in the RNA splicing (e.g., SF3B1, SRSF2, U2AF1, ZRSR2, LUC7L2, DDX41) and epigenetic modifications, such as histone modification (e.g., ASXL1, EZH2) and DNA methylation (e.g., TET2, DNMT3A, IDH1/IDH2) [3, 119]. TP53 mutation predicts significant adverse clinical outcomes in MDS, however, clinical implications of other gene mutations remain unclear [119]. Recently, a lot of genomic information has been discovered in patients with AML and MDS, such as gene mutation with specific type of AML, genomic overlapping across different categories, even with the age-related frequency of selected recurring gene mutations [113]. However, the pathogenesis of AML caused by these gene mutations has yet to be further explored.

## Conclusion

New understanding about the recurrent gene mutations in AML patients has been achieved regarding its clinical implications in the past years. Three recurrent gene mutations (RUNX1, ASXL1, and TP53) have been added in the risk stratification of the ELN-2017 recommendations for AML [2]. Recently, more novel fusion genes, such as GTF2I–PDGFRB and IKZF1–TYW1 fusion genes, and PAN2/PAN3 complex, have reported and may be clinically important [120, 121]. As the new technology advances, more recurrent gene mutations will be explored and used for AML risk stratification and treatment guidelines. Furthermore, gene mutation-targeted new drugs and therapies will be developed and benefit more AML patients in the near future.

## Data Availability

Not applicable.

## Abbreviations

AML: Acute myeloid leukemia
AR: Allele ratio
ASXL1: Additional sex combs-like 1
biCEBPA: Biallelic mutations of CCAAT/enhancer binding protein alpha
CEBPA: CCAAT/enhancer binding protein alpha
CHIP: Clonal hematopoiesis of indeterminate potential
CR1: First complete remission
DNMT3A: DNA methyltransferase 3A
EFS: Event-free survival
ELN: European Leukemia Net
FLT3: FMS-like tyrosine kinase 3
FLT3-ITD: FLT3 mutations: internal tandem duplication
FLT3-TKD: FLT3 mutations: tyrosine kinase domain
IDH: Isocitrate dehydrogenases
KMT2A: Lysine (K)-specific methyltransferase 2A
moCEBPA: Monoallelic mutations of CCAAT/enhancer binding protein alpha
MRD: Minimal residual disease
NGS: Next-generation sequencing
NPM1: Nucleophosmin
OS: Overall survival
R/R: Relapsed/refractory
RUNX1: Runt-related transcription factor 1
STAT5: Signal transducer and activator of transcription 5
TP53: Tumor protein p53
VAF: Variant allele frequency
